# “Body & Brain”: effects of a multicomponent exercise intervention on physical and cognitive function of adults with dementia - study protocol for a quasi-experimental controlled trial

**DOI:** 10.1186/s12877-021-02104-1

**Published:** 2021-03-04

**Authors:** Joana Carvalho, Flávia Borges-Machado, Duarte Barros, Arnaldina Sampaio, Inês Marques-Aleixo, Lucimere Bohn, Andreia Pizarro, Laetitia Teixeira, José Magalhães, Óscar Ribeiro

**Affiliations:** 1 Faculdade de Desporto da Universidade do Porto, Rua Dr. Plácido Costa 91, 4200-450 Porto, Portugal; 2 CIAFEL, Centro de Investigação em Atividade Física, Saúde e Lazer, Universidade do Porto, Rua Dr. Plácido Costa 91, 4200-450 Porto, Portugal; 3 Faculdade de Educação Física e Desporto, Universidade Lusófona, Rua de Augusto Rosa 24, 4000-098 Porto, Portugal; 4 Instituto de Ciências Biomédicas Abel Salazar, Universidade do Porto, Rua Jorge de Viterbo Ferreira 228, 4050-313 Porto, Portugal; 5 CINTESIS, Centro de Investigação em Tecnologias e Serviços de Saúde, Departamento de Educação e Psicologia, Universidade de Aveiro – Campus Universitário de Santiago, 3810-193 Aveiro, Portugal

**Keywords:** Neurocognitive disorder, Multimodal, Functionality, Cognition

## Abstract

**Background:**

Dementia is a leading cause of death and disability that was declared as one of the greatest health and social care challenges of the twenty-first century. Regular physical activity and exercise have been proposed as a non-pharmacological strategy in disease prevention and management. Multicomponent Training (MT) combines aerobic, strength, balance and postural exercises and might be an effective training to improve both functional capacity and cognitive function in individuals with dementia (IwD). Nevertheless, data on the effects of MT in IwD are still limited and the extent to which IwD can retain improvements after an exercise intervention still needs to be elucidated. The aim of “Body & Brain” study is to investigate the effects of a 6-month MT intervention and 3-month detraining on the physical and cognitive function of IwD. Additionally, we aim to explore the impact of this intervention on psychosocial factors and physiologic markers related to dementia.

**Methods:**

This study is a quasi-experimental controlled trial using a parallel-group design. The study sample consists of community-dwelling individuals aged ≥60 years who are clinically diagnosed with dementia or major neurocognitive disorder. Participants will be either allocated into the intervention group or the control group. The intervention group will participate in MT biweekly exercise sessions, whereas the control group will receive monthly sessions regarding physical activity and health-related topics for 6 months. The main outcomes will be physical function as measured by the Short Physical Performance Battery (SPPB) and cognitive function evaluated using the Alzheimer Disease Assessment Scale – Cognitive (ADAS-Cog) at baseline, after 6-months and 3-months after the end of intervention. Secondary outcomes will be body composition, physical fitness, daily functionality, quality of life, neuropsychiatric symptoms and caregiver’s burden. Cardiovascular, inflammatory and neurotrophic blood-based biomarkers, and arterial stiffness will also be evaluated in subsamples.

**Discussion:**

If our hypothesis is correct, this project will provide evidence regarding the efficacy of MT training in improving physical and cognitive function and give insights about its impact on novel molecular biomarkers related to dementia. This project may also contribute to provide guidelines on exercise prescription for IwD.

**Trial registration:**

ClinicalTrials.gov – identifier number NCT04095962; retrospectively registered on 19 September 2019.

## Background

Dementia is considered one of the main age-related health problems impacting the modern society [1, 2]. Also designated as major neurocognitive disorder, these umbrella terms are used to describe a set of diseases that are mostly chronic or progressive, affecting the brain and several cognitive functions [3, 4]. By 2050, it is expected that 132 million people will suffer from dementia worldwide [1]. Therefore, the World Health Organization (WHO) has already recognized this condition as a global health priority [5] and alerted countries for the need to develop and implement national intervention plans to prevent, treat and care for dementia [6]. In Portugal, the prevalence rate for dementia in 2017 was 9.23% in community-dwelling older adults [7].

Age is the primary non-modifiable risk factor for dementia [8]. Livingston et al. (2020) described 12 potentially modifiable risk factors that, when addressed might prevent or delay up to 40% of dementia cases [2]. Of those, addressing physical inactivity (e.g., by increasing the levels of physical activity), particularly in later life, may be a potential strategy to prevent, or even delay the progression of cognitive impairment due to its effect on underlying physiologic mechanisms: the indirect effects on general cardiovascular health/fitness and neurological direct effects [9–11].

Individuals diagnosed with dementia (IwD) not only exhibit a significant decline in one or more cognitive domains that interfere with the person’s ability to perform activities of daily living (ADL), but also tend to reveal increased declines on physical fitness, as impaired aerobic capacity, muscle strength, agility, gait and balance [12–14]. Skinner, Ellis & Pa (2018) stated that exercise may positively impact IwD’s cognition, quality of life, functional status, sleep, mood and physical function [11], being these positive outcomes related to several cellular and molecular alterations [15]. Additionally, evidences suggest that exercise can counteract dementia-related pathogenic changes by preserving neuroplasticity trough neurogenesis, synaptogenesis and angiogenesis [15]. Hence, increasing data advocate regular moderate-intensity exercise as an effective strategy to improve general health status, reducing vascular risk factors, obesity, levels of inflammatory markers, improving metabolism and brain health in older adults with dementia [16]. However, results regarding exercise effects on cognitive function are still heterogeneous [17–19].

Although exercise interventions for IwD seem to be feasible and well tolerated, resulting in positive effects on ADL functionality for people at mild-to-moderate stages [17, 18], the therapeutic role of physical activity, particularly exercise, after dementia diagnosis still needs further evidence [20–22], − especially when considering community-based contexts and caregivers as participants on exercise sessions [23]. It also matters to highlight that the dose-dependent relationship remains unclear [11, 18, 24]. Therefore, research is needed to identify the triad: stage/type of dementia, FITT variables (frequency, intensity, type and time) of exercise intervention, and target outcome [11, 25].

Regarding exercise modality, Multicomponent Training (MT) [26] – combining aerobics, strength and balance exercises – seems to be effective at improving functional and cognitive performances in older adults with neurodegenerative disorders, particularly dementia [27–29]. Exercise programs previously developed by our group confirmed that a 6-month MT intervention can positively impact the physical and cognitive function of institutionalized older adults with Alzheimer's Disease (AD) [30, 31] and can be beneficial in physical fitness and ADL functionality performance among community-dwelling patients [25, 32]. However, the extent to which IwD can retain these improvements after the cessation of MT intervention still need to be elucidated [33] in order to understand how detraining affects functionality and cognition following the cessation of MT stimulus.

In addition to the most common analysis on cognition, physical fitness, daily functionality, neuropsychiatric symptoms, quality of life and caregivers’ burden [18, 34–36], several other parameters need to be further explored when considering the benefits of MT. These include the effect of exercise on body composition, cardiorespiratory fitness, vascular, inflammatory and neurotrophic blood-based biomarkers, and arterial stiffness.

The primary purpose of this study is to investigate the effects of a 6-month MT exercise intervention and 3-months of detraining on physical and cognitive function of older adults diagnosed with dementia. Secondarily, we aim to explore potential positive effect of MT exercise on IwD psychosocial and physiological outcomes. We hypothesize that exercise positively modulates body composition (e.g., by increasing lean mass and decreasing adiposity) and therefore may improve physical fitness and function. Additionally, it is described that exercise enhances cerebrovascular plasticity, peripheral biomarkers associated with brain blood flow and (neuro)inflammation, consequently attenuating cognitive decline or even improving cognitive function. The concomitant enhancements of physical and cognitive function in individuals with dementia may help to preserve or improve their functionality in ADL and manage behavioral and psychological symptoms of dementia (BPSD), reducing the caregiver’s burden and leading to improvements in quality of life.

## Methods and analysis

The “Body & Brain” study is registered with the US National Institutes of Health clinical trials registry (ClinicalTrials.gov – identifier number NCT04095962). This protocol report complies with the Standard Protocol Items: Recommendations for Interventional Trials (SPIRIT) 2013 checklist: recommended items to address in a clinical trial protocol and related documents [37].

### Trial design

This study is a multicenter quasi-experimental controlled trial with a parallel design, that will be conducted in a wide-ranging public and private settings (e.g., daycare centers and local community centers) in the Porto metropolitan area, Portugal. Participants will be recruited from community programs, daycare centers, psychiatric hospitals and clinics (i.e., outpatients followed within psychiatry or neurology specialists), Alzheimer’s and caregivers’ associations, municipalities, local journals, and social media. Advertisements will be made via phone calls, emails, informative flyers and presential and online meetings. In order to motivate and clarify concerns about the exercise sessions, a short MT session will be provided to exemplify and illustrate a typical session for individuals with dementia.

**Fig. 1:**
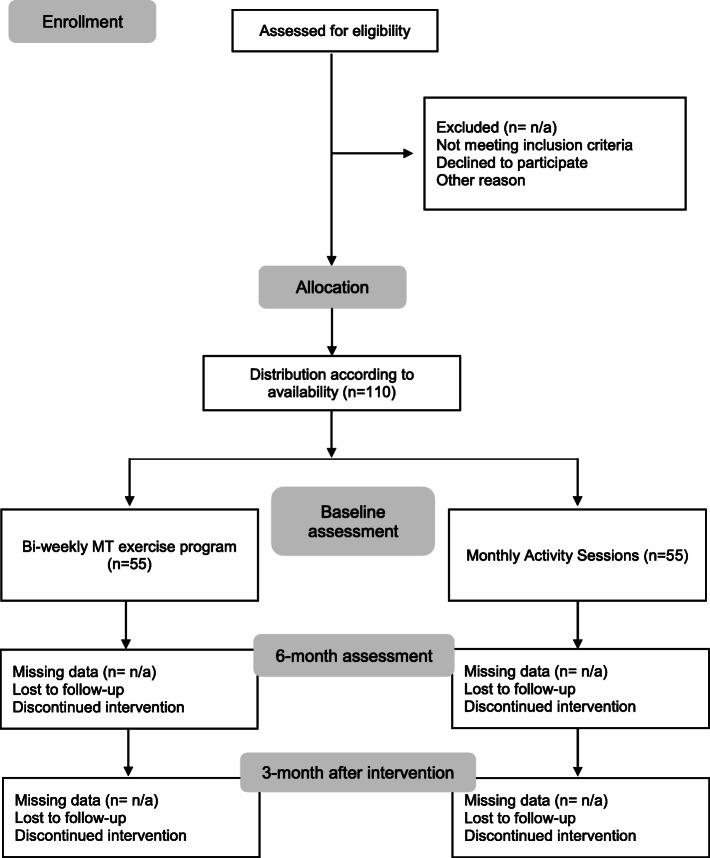
Study Flow Diagram

Eligible participants and caregivers will receive a complete explanation of the study purposes, risks and procedures. Following the best ethical procedures, informed consent will be obtained from interested dementia participants and from their main caregivers/legal representatives/significant person. After agreeing to participate and according to personal availability, participants will be allocated into a 6-month MT intervention (IG) or into a social activity group (CG) (see Fig. 1). All CG participants will be offered the possibility to participate in an exercise program after the end of the study cycle.

Participants will be assessed at baseline, after 6-month of intervention, and 3-month follow-up after the end of intervention (Table 1). At baseline, sociodemographic (e.g., age and years of formal education), general clinical data (e.g., presence of comorbidities, pharmacological treatment, and falls) will be collected through a structured questionnaire, and dementia rating severity evaluation will be performed trough the Clinical Dementia Rating (CDR) outcome measurement [38]. During baseline assessments, participants will not be informed regarding to which group they belong. Data collection will be performed by well-trained and experienced researchers.

**Table 1:** Description of methods and/or instruments to evaluate IwD and/or their caregivers/significant person/proxy decision-maker

	Domain	Measure	Data from	
			IwD	Caregiver
**Screening**	Informed Consent		X	X
	Anamnesis (sociodemographic and general clinical data)			X
	Clinical Dementia Rating		X	X
**Primary**^**a**^	Cognition			
	Cognitive Function	ADAS-Cog	X	
	Physical Fitness			
	Lower Limb Function	SPBB	X	
**Secondary**^**a**^	Cognition			
	Cognitive Function	MMSE	X	
		TMT		
	Physical Fitness			
	Cardiorespiratory Capacity	Modified Bruce Treadmill Test	X	
	Physical Fitness	SFT	X	
	Static Balance	One Leg Balance Test	X	
	Handgrip Strength	Handgrip Dynamometer	X	
	Body Composition and Anthropometry			
	Body Mass	DXA (Hologic QDR 4500, Explorer model, version 12.4)	X	
	Total Fat-free Mass			
	Fat Mass			
	Appendicular Skeletal Mass Index			
	Weight	Weighting Scale	X	
	Height	Stadiometer	X	
	Waist and Hip Circumferences	Measuring Tape	X	
	Quality of Life and ADL’s Functionality			
	Basic and instrumental ADL	Lawton & Brody IADL Scale		X
		Barthel Index		
	Quality of Life	QoL-AD	X	X
	Behavioral and Psychological Symptoms of Dementia			
	Neuropsychiatric Symptoms and Caregiver Distress	NPI		X
	Caregiver Burden			
	Caregiver Well-being and Subjective Burden	CarerQol-7D		X
		CarerQol-Vas		
	Blood-based Biomarkers and Hemodynamics			
	Systolic and Diastolic Blood Pressure	Digital Sphygmomanometer	X	
	Arterial Stiffness	Pulse Wave Velocity (SphygmoCor, AtCor Medical, Australia)	X	
	TG	Concentration in blood plasma	X	
	TC			
	LDL-C			
	HDL-C			
	HbA1c	HPLC		
	BDNF	ELISA	X	
	VEGF			
	CRP			
	sICAM-1			
	sVCAM-1			
	MMP-9			
	IL-6, IL-8, IL-10			
	TNF-alfa	CLIA	X	
	IGF-1			
**Confounders**^**a**^	Daily Physical Activity			
	Physical Activity Levels	Accelerometer-based activity monitors *GT9X+ Link* (ActiGraph)	X	

^a^*T0* Baseline, *T1* After 6-month intervention, *T2* 3-month follow-up

The same evaluator will be responsible to perform the same procedures along the three assessment periods, which will be subdivided in five separate moments (Table 2). The exercise professional who is going to provide the MT sessions will not be involved in any data collection.

**Table 2:** Schedule for the different primary and secondary and outcomes, screening, and confounder evaluations of each assessment moment per evaluator

*Assessment moments per evaluator*	Prior to Assessment (only at baseline)	1st Cognition & Functionality^**a**^	2nd Cognition, QoL & Physical Fitness^**a**^	3rd Physical Activity Level^**a**^	4th ADL, QoL, BPSD & Caregiver Burden^**a**^	5th Body Composition, Cardiorespiratory Fitness, Hemodynamics & Blood-based Biomarkers^**a**^
***Evaluator 1***	CDR	MMSE TMT	ADAS-Cog QoL-AD patient		QoL-AD caregiver Carer-QoL NPI Barthel Index Lawton & Brody IADL Scale	
***Evaluator 2***		SPPB Handgrip strength	SFT One Leg Balance Test	7-day accelerometer-based monitor		
***Evaluator 3***	Informed Consent and anamnesis (sociodemographic and general clinical data)					VO2 Peak Consumption Anthropometry DXA Body Composition Blood Pressure Arterial Stiffness
***Laboratory***						Blood Analysis

^a^*T0* Baseline, *T1* After 6-month intervention, *T2* 3-month follow-up

### Participants

#### Eligibility criteria

This study will include individuals aged ≥60 years capable of walking autonomously without an assistive device or human assistance, and diagnosed with dementia or major neurocognitive disorder using accepted diagnostic criteria such as that established by the Diagnostic and Statistical Manual of Mental Disorders (DSM-IV-TR or DSM-5) [3], ICD-10 [39], or the NINCDS-ADRDA [40]. Potential participants must have been diagnosed by a physician for at least for 6-months. Participants diagnosed with Parkinson’s disease, Frontotemporal disease, Lewy bodies’ disease, Huntington’s disease or other rare forms of dementia can be included in the study as long as they do not exhibit significant motor/functional limitations. The exclusion criteria comprise disorders or conditions in which exercise is contraindicated such as unstable or ongoing cardiovascular and/or respiratory disorder; hospitalized individuals and/or recovering from surgery or rehabilitation; and presenting an advanced stage of dementia (e.g., scored 3-points in CDR or ≤ 10 points on MMSE) that could affect physical performance in the exercise training sessions or testing procedures.

#### Sample size

Sample size was estimated based on ANCOVA for the analysis of the differences [41]. To detect an adjusted 1 point (SD = 2.1) difference in SPPB after intervention between IG and CG with 80% power and an alpha level of 0.05, a total of 110 participants (55 participants per group) will be needed, already accounting for an estimated dropout of 25%. Sample size calculation was performed using G*Power 3.1.3 (Universität Düsseldorf, Düsseldorf, Germany) [42].

### Study intervention

#### Experimental group: exercise training

The MT program will be conducted for 6 months, twice a week in 60 min sessions. Sessions will be divided in warm-up (10 min, including slow walk, postural and mobility exercises for general activation, and stretching exercises), specific training (35–45 min, including balance/coordination training, strength and aerobic exercises) and cool down (5 min with breathing and stretching exercises for the main worked joints and muscles) following the main guidelines recommended by the American College of Sports Medicine [43] and the WHO [44] (Table 3).

**Table 3:** Exercise intervention plan for “Body & Brain” study

Warm-up	Balance & Coordination		Strength		Aerobic Resistance	Stretching & Cool down
General activation, joint mobilization, stretching exercises	Static and dynamic balance; coordination exercises		Strength exercises involving mainly major upper and lower body muscle groups		Low impact aerobic exercises involving walking, stationary march and dance	Lower body and upper body stretch, breathing exercises
e.g. slow walk, mobility exercises for the shoulders, trunk, hip, and ankles as rotations, flexion/extension and adduction/abduction	e.g. one leg stand, tandem walk, sideways walk, heel-to-toe walk, step over objects as cones, hoops and huddlers, exercises with balance pads, shift weight from foot to foot, turning and changing direction		e.g. biceps arm curl, triceps extension, adapted planks, seated row, chest press, squats, knee extension, hip extension, toe raises, standing leg curl		e.g. stationary marching, walking, simple step-based choreographic movements - exercises will start with only lower limbs and as needed will include upper limbs	e.g. back and chest stretch, quadriceps and hamstrings stretch, diaphragmatic breathing
**1st & 2nd months**						
10 min	15 min	2–3 exercises	15 min	4 exercises	10 min (5 + 5)	5 min
	Dynamic balance		2 × 10–12 reps		60–65% HRmax	
**3rd month**						
10 min	15 min	2–3 exercises	15 min	4–6 exercises	10 + 5 min	5 min
	Dynamic balance and coordination		2 × 10–12 reps		65–75% HRmax	
**4th month**						
10 min	10 min	2–3 exercises	15 min	4–6 exercises	10 + 10 min	5 min
	Dynamic balance and coordination		2 × 8–10 reps		65–75% HRmax	
**5th and 6th months**						
10 min	10 min	2–3 exercises	15 min	4–6 exercises	10 + 10 min	5 min
	Dynamic balance and coordination		2 × 8–10 reps		65–80% HRmax	

HRmax *Maximum heart rate,* min *Minutes,* reps *Repetitions*

Well-designed, routine, simple, enjoyable and functional exercises will be preferred. A 1 month adaptation period, before the training program, will be implemented with the main purpose of promoting familiarization with exercises and socialization between participants [45]. New exercises should be introduced based only as a progression of previous well-known exercises. In the adaptation period the focus will be in learning the movements keeping a low intensity, of 40 to 50% of HRmax in the aerobic exercises and 1 set of 12 to 15 slow repetitions for the strength exercises.

Exercise prescription, implementation and evaluation will be performed by specialized exercise professionals. Furthermore, prior to the program implementation, those professionals will receive specific training concerning dementia (clinical features, signs and symptoms), challenging behaviors, communication strategies, and other related topics (e.g., safety issues) [46].

Sessions, involving 5 to 12 participants will be held in appropriate spaces and in a safe and calm environment to limit distractions and maximize the individuals’ participation [46]. Sessions are going to be scheduled in the late morning or in early afternoon period, and the sports equipment encompasses chairs, ground markers, strings, elastic resistance bands, dumbbells, hurdles, balance pads, balls, and steps. Whenever possible, sessions will be accompanied by music, particularly during the aerobic part, in order to confer appropriate exercises intensity and enthusiasm [45].

Balance exercises will gradually reduce the base of support and/or reduced sensory input as well as include dynamic movements that perturb the center of gravity. These progressions will occur when participants reach the time limit without losing control of the body. Also, focus on some easy coordination exercises and conscious control of the body will be performed.

Four to 6 multi-joint strength exercises involving major muscle groups will be included in each session. The number of repetitions will decrease from 10 to 12 to 8–10 with increasing load that could be lifted correctly to volitional fatigue. A rest period of 1′30’-2″ will be completed between each set.

Aerobic endurance will be attained with low impact exercises at 60–65% of HRMax implemented in two 5-min periods. Intensity will increase progressively with increasing duration and HR until reaching 2 periods of 10 continuous minutes of exercise at 75 to 80% of HRmax. Sessions will be monitored using heart rate monitors [47], and whenever possible, the Modified Borg Scale of Perceived Exertion will be applied.

Exercise professionals will registry adverse events (such as pain or fatigue) and number of falls during exercise sessions to provide information on safety of the MT program. Finally, attendance to sessions will be recorded and reasons for absences the will be catalogued [48].

Although this is not a dyadic intervention, caregivers will be included in each session as class members, but focus will be directed to individuals diagnosed with dementia. Along with the novelty of this strategy, this approach will facilitate dementia patients’ participation once their transportation, motivation, and involvement are dependent of their caregivers [49, 50]. Additionally, caregivers’ participation will also be determinant to deal with behavioral and psychological symptoms of dementia patients [46].

#### Control group: social activity

Participants in the control group will receive monthly sessions regarding physical activity and health-related topics as a complement to standard care. No specific exercise intervention will be conducted for this group. Due to ethical reasons, there will be no limit on co-interventions during the trial. Pharmacological, medical or other types of treatments may be initiated, continued or discontinued independently of research team approval. However, participants who eventually are going to participate in further co-interventions will be signalized, and information on type and duration will be collected and considered as covariates in statistical procedures. Participants are going to be contacted with a regular basis via phone calls to assure retention and motivation.

## Outcomes

### Primary outcome

Our primary outcomes are physical and cognitive function. Physical function will be measured using SPPB [51, 52] that assesses balance, gait capacity and lower limb strength. The total score ranges from 0 to 12, with higher scores indicating better function. A 1-point change has been described as a meaningful change in physical performance [42]. Cognitive function will be evaluated using the Portuguese version of the ADAS-Cog [53, 54] which comprises fundamental features of cognitive decline such as memory, praxis, constructive ability, language and orientation. Scores range from 0 to 68, with higher scores suggesting greater severity of cognitive impairment. A 4-point ADAS-Cog change has been described as clinically meaningful [55, 56]. As presented on Table 1, primary and secondary outcomes are going to be measured at baseline, after 6-month of intervention, and 3-month follow-up after the intervention has ended.

### Secondary outcomes

#### Physical fitness

Physical fitness is going to be measured via the Senior Fitness Test (SFT) [57]. The SFT is reliable for assessing physical fitness in older adults (≥ 60 years old) including those with cognitive impairment [58, 59]. The SFT includes lower and upper-body strength (chair-stand and arm curl test, respectively), aerobic endurance (2-min steps test), and agility/dynamic balance (8-foot up-and-go test). Static balance will be measured with the One Leg Balance Test [60, 61] which has been considered a reliable for IwD. Oxygen consumption (VO_2_ peak) will be measured trough an incremental treadmill test using a modified Bruce protocol designed for older individuals, previously tested with participants diagnosed with AD [62, 63] and which reliability at mild stages has been proved [64]. Finally, handgrip strength will be obtained with a Jamar hand-dynamometer [65], commonly used in IwD.

#### Cognitive function

The Mini Mental State Examination (MMSE) [66, 67], a widely used test of cognitive function among older adults will be used and consists of a 30-item instrument (with higher scores indicating better cognitive performance) that is organized in six cognitive domains – orientation, retention, attention and calculation, delayed recall, language, and visuo-constructive ability. Frequently used in this research field, MMSE test results will allow comparisons between different studies. As stated on *Bossers* et al. (2012) systematic review study, the MMSE and ADAS-Cog are the most used outcome measures to evaluate global cognitive functioning in clinical trials with older adults with dementia. In addition, these tests were found to be valid and reliable in patients with different subtypes of dementia (i.e., Alzheimer’s disease, vascular disease and Lewy body disease) from mild to moderate stages [68]. Executive function will be assessed with the Trail Making Test (TMT) [69] part A – attention, visual scanning, speed of eye-hand coordination and information processing; and part B – working memory and the ability to switch between different stimuli. Time and number of errors will be registered. This test is highly related to participants ability to perform instrumental ADL, and has been used in similar studies [70, 71].

#### ADL’s functionality

Participants independence on performing ADL will be assessed with widely used measurements: the Barthel Index [72, 73] to address ten basic daily activities, such as bathing, dressing and using toilet – with total score ranging from 0 to 100; and the Lawton & Brody Instrumental Activities of Daily Living (IADL) scale [74] – total score ranging from 0 to 8 – to address more complex activities, as shopping, food preparation and ability to handle finances. Lower scores indicate higher problems to perform activities, within both instruments.

#### Body composition & antropometrics

Body composition (e.g., body mass, fat-free mass, fat mass, and appendicular skeletal mass index) will be analyzed through DXA (QDR 4500/A, Hologic Explorer, Bedford, USA) – previously used with AD participants [75]. Anthropometric measurements will be taken using standardized protocols. Waist and hip circumference will be assessed at the midpoint between iliac crest and the bottom of the ribcage, and at the widest portion of the buttocks, with the tape parallel to the floor, respectively, using a spring-loaded measuring tape.

#### Quality of life & BPSD

The Quality of Life – Alzheimer’s Disease Scale (QoL-AD) [76] will be used to measure the dementia participants’ quality of life. The questionnaire includes 13 items such as physical health, energy, mood, memory, family, marriage, friends, and ability to do things for fun. The QoL-AD provides the participant and the caregiver reports of the participant’s QoL and is scored on a 4-point Likert-type scale ranging from 1 to 4 (excellent), with total scores ranging between 13 and 52 points. The Neuropsychiatric Inventory [77] will be used to determine the frequency and severity of BPSD with a total score ranging from zero to 144 points with high scores corresponding to worsening of BPSD.

#### Caregivers burden

The Care-related Quality of Life instrument (CarerQol) will be used to address subjective burden (CarerQol-7D), and caregivers’ well-being (CarerQol-VAS), using a visual analogue scale to ask about happiness between 0 and 10 (completely happy) [78, 79]. The subjective burden is measured in seven dimensions: fulfillment, relational problems, mental health, daily activities problems, physical health and support. Total CarerQol-7D score ranges from 0 to 14 points with higher scores indicating better caregiving situation.

### Subsample studies

#### Blood-based biomarkers and hemodynamics

At baseline, after the intervention (6-months) and 3-months after intervention has ended, venous blood samples from the antecubital vein will be taken from a subsample. All the biochemical analysis will be performed in a certified commercial laboratory.

Non-fasting venous blood samples will be collected into serum-separating tubes (serum isolation) or tubes containing EDTA (whole blood or plasma isolation). Traditional plasma biochemical parameters, including triglycerides (TG), total cholesterol (TC), high-density lipoprotein cholesterol (HDL-C) will be enzymatically measured [80]. Low density lipoprotein-cholesterol (LDL-C) will be determined according to the Friedewald formula estimated by subtracting HDL-C and one fifth of the triglyceride value from the total cholesterol level [81]. Whole blood glycated hemoglobin (HbA1c) will be measured using high performance liquid chromatography (HPLC) [82].

Serum levels of C-reactive protein (CRP, high-sensitivity test), intercellular adhesion molecule-1 (sICAM-1), vascular cell adhesion molecule-1 (sVCAM-1), metalloproteinase-9 (MMP-9), interleukin (IL) 10, IL-8, IL-6, brain-derived neurotrophic factor (BDNF) and vascular endothelial growth factor (VEGF) will be evaluated by an enzyme-linked immunosorbent assay (ELISA) using commercially assay kits according to the manufacturer’s instructions (R&D System, Minneapolis, MN, USA). Serum levels of creatinine will be measured using the Jaffé-method [83]. Serum levels of tumor necrosis factor-alfa (TNF-alfa) and insulin like growth factor 1 (IGF-1) will be analyzed by chemiluminescent immunoassay (CLIA) method accordingly to the manufacturer’s protocol (Siemens Immunolite 2000, Munich, Germany).

These blood biomarkers will be included in the project due to current evidence of their involvement with dementia risk factors, such as dyslipidemia, inflammation, and endothelial dysfunction [84]. Additionally, we will evaluate blood biomarkers known to play an important role in the relationship between exercise and brain health, including the growth factors BDNF, IGF-1 and VEGF [85].

The same subsample will be assessed for blood pressure and arterial stiffness. Blood pressure is going to be assessed by a digital sphygmomanometer (Colin, BP 8800, Critikron, Inc., USA) and arterial stiffness is going to be measured as carotid-femoral pulse wave velocity (cfPWV) using applanation tonometry (SphygmoCor, AtCor Medical, Australia). Procedures will follow international guidelines [86]. In brief, sequential and consecutive carotid and femoral pressure waves are going to be registered together with the electrocardiogram, that will serve as a reference to calculate the transit time between the recording’s sites. The distance travelled by the pressure wave will be the direct distance between the recording points at the femoral and carotid arteries, corrected by the factor 0.8 [86]. The value of cfPWV is going to be calculated as the direct distance (in meters) divided by the transit time (in seconds). All measurements will be performed in duplicate by the same trained researcher.

### Confounders

Habitual physical activity levels will be collected before, after the 6-month intervention and at 3-month follow-up using an accelerometry-based method – *ActiGraph GT9X Link* -, for 7 consecutive days [87]. Participants will be instructed to wear the accelerometer attached to an elastic belt at all times except when sleeping, bathing, swimming or other water activities. Data will be collected in 100 Hz epochs and a minimum of 10-h on at least 4 days (1 weekend) will be considered valid data. Physical activity data will be processed using *Actilife* software (*Actigraph LLC* Pensacola, FL) and summarized as time spent in sedentary, light, moderate to vigorous physical activity.

### Statistical analysis plan

Descriptive statistics will be expressed as mean and standard deviation (SD) or as median and interquartile range (IQR) for continuous variables. Categorical variables will be expressed as frequency and percentages. Normal distribution will be analyzed by the Kolmogorov-Smirnov test with Lilliefors’ (K–S) significance correction and normal probability plots. Statistical comparisons at baseline characteristics will be performed using t-test or Mann–Whitney U-test for continuous variables and Chi-squared test or Fisher’s test for categorical variables.

Age, gender, level of education, dementia type/stage, and other variables will be considered as potential confounders and adjustments will be performed in accordance to detect differences between experimental and control group.

The primary effect parameter will be the difference in the SPPB and ADAS-Cog tests between the experimental and control groups, considering the two different moments of evaluation (baseline and after 6-month of intervention). An analysis of variance (ANOVA) test with repeated measures adjusted for potential confounders will be performed for the intervention-related effects. When a significant F value is obtained, Bonferroni post hoc procedures will be used to evaluate pairwise differences. Additionally, mixed effects models will be performed in order to identify potential predictive factor associated with the changes in primary and secondary outcomes from baseline, post-intervention and over the 3-months detraining period.

A multivariable regression analysis will be performed in order to explore the association between cardiovascular and blood-markers, and cognition.

Statistical analyses will be conducted with the SPSS IBM Statistical Software version 25.0 (SPSS, Inc., Chicago, IL) for Windows with a significance level of *p* < 0.05.

### Data management and confidentiality

An identification code will be attributed to each participant with common designation for IG and CG. The correspondence list between name and ID will be stored in a lockable cabinet at the Principal Investigator office located at the Research Centre, and separately of the report forms and evaluation instruments. Any personal information such as name, address or other identification data will not be collected nor registered. Data will be entered into an electronic database by two authorized researchers. The management and any statistical treatment will be only conducted after the authorization of the Principal Investigator. Data will be destroyed after 10 years of the end of intervention, and electronic data will be deleted 5 years after the last scientific publication.

### Ethics and dissemination

The study protocol was approved by the Ethical Committee of the Faculty of Sports of the University of Porto (Ref CEFADE22.2018). All participants and caregivers/legal representatives/significant person will be asked to sign an informed consent. Data confidentiality and anonymity will be guaranteed in all phases of the study. All procedures performed will follow the ethical standards of the institutional and/or national research committee and the 1964 Helsinki Declaration and its later amendments or comparable ethical standards. We intend to disseminate study results via presentations in national and international conferences and manuscripts in peer reviewed scientific journals. Additionally, we will promote seminaries directed for the entire community and especially for the participants, caregivers, health care professionals and organizations. We also intend to disseminate important results through local public media. Participants and caregivers will be informed individually of study results via synthetized reports.

## Discussion

This study describes the protocol of a quasi-experimental controlled trial of a 6-month MT intervention for IwD comprehended on “Body & Brain” project. Some innovative aspects of this trial are worthy highlighting. For instance, we believe that tailored, individually adjusted, and sustained MT led by a well-trained exercise specialist, may positively impact physical and cognitive function of people living with dementia, independently of their type/stage of dementia. Another innovative aspect of this trial will be the inclusion of caregivers in the exercise sessions. This is of particular relevance for improving the dynamic relationship between caregiver and IwD, to increase participants’ adherence to the intervention and the caregivers’ awareness regarding positive nonpharmacological interventions in dementia care [88]. Thus, by including caregivers in exercise sessions, we expect that both the caregiver and the care-recipient will remain active after the end of the trial. This intervention may also positively impact IwD neuropsychiatric symptoms, and therefore improve quality of life. Informal caregivers’ quality of life and ability to cope with BPSD’s may also be improved, especially if they exchange experiences with other caregivers. Finally, at a molecular level, we aim to examine how exercise can positively impact the neurotropic and inflammatory factors, as well as the endothelial function, commonly altered in IwD [89]. Our findings will give much needed insights about its impact on the pathophysiology of dementia and help to improve the current evidence regarding exercise as non-pharmacological intervention in IwD.

Some challenges in this trial must be recognized. First, due to the study design and the dementia paradigm in the Portuguese population, we anticipate a heterogeneous sample, under(/mis) diagnosed and with many different comorbidities [90, 91]. Secondly, IwD tend to be sedentary for most of the day and practice low-intensity physical activities [92], which is particularly concerning in the Portuguese paradigm, where older population are highly sedentary [93], Therefore, authors expect some difficulties during recruitment. Nevertheless, this project is expected to raise awareness on exercise intervention for IwD among caregivers, institutions that provide care services for older adults, policy makers and general community. We expect to contribute to the cultural change regarding the therapeutic options to manage dementia. Moreover, our goals goes along with the WHO recommendations for the functional health era in which exercise has a decisive role [4]. Finally, presuming that our data will show that IwD can respond positively to a moderately challenging tailored MT exercise, our project can be a significant step to move towards in what concerns older adults exercise prescription. Authors believe that this research may contribute to disseminate and generalize exercise prescription among this population in order to prevent disability and attenuate or delay the cognitive decline. As long as the cure for dementia remains to be achieved, and the pharmacological limitations of treatment remain, we cannot neglect the global and coadjuvant role MT exercise may have as a non-pharmacological treatment.

### Trial status

The trial commenced recruitment in September 2018 and is currently in process. Recruitment will cease when the expected number of participants has been achieved.

## Data Availability

Data sharing is not applicable to this article as no datasets were generated or analyzed during the current study.

## Abbreviations

AD: Alzheimer’s Disease
ADAS-Cog: Alzheimer’s Disease Assessment Scale - Cognitive
ADL: Activities of Daily Living
ANOVA: Analysis of Variance
BDNF: Brain-derived Neurotrophic Factor
BPSD: Behavioral and Psychological Symptoms of Dementia
CarerQol: Care-related Quality of Life instrument
CDR: Clinical Dementia Rating
cfPWV: Carotid-femoral Pulse Wave Velocity
CG: Control Group
CLIA: Chemiluminescent Immunoassay
CRP: C-reactive Protein
DSM-5: Diagnostic and Statistical Manual of Mental Disorders Fifth Edition
DSM-IV-TR: Diagnostic and Statistical Manual of Mental Disorders Fourth Edition – Text Revision
DXA: Dual Energy X-ray Absorptiometry
ELISA: Enzyme-linked Immunosorbent Assay
HbA1c: Glycated Hemoglobin
HDL-C: High-Density Lipoprotein Cholesterol
HPLC: High Performance Liquid Chromatography
HRmax: Maximum Heart Rate
ICD-10: International Statistical Classification of Diseases and Related Health Problems-10th Revision
IG: Intervention Group
IGF-1: Insulin Like Growth Factor 1
IL-6, IL-8, IL-10: Interleukin 6, 8, 10
IwD: Individuals with Dementia
IADL: Instrumental Activities of Daily Living
IQR: Interquartile Range
LDL-C: Low-Density LipoproteinCholesterol
MMP-9: Metalloproteinase-9
MMSE: Mini Mental State Examination
MT: Multicomponent Training
NINCDS-ADRDA: National Institute of Neurological and Communicative Disorders and Stroke - Alzheimer’s Disease and Related Disorders Association
NPI: Neuropsychiatric Inventory
QoL-AD: Quality of Life-Alzheimer’s Disease
SD: Standard Deviation
SFT: Senior Fitness Test
sICAM-1: Intercellular Adhesion Molecule-1
SPPB: Short Physical Performance Battery
sVCAM-1: Vascular Cell Adhesion Molecule-1
TC: Total Cholesterol
TG: Triglycerides
TMT: Trail Making Test
TNF-alfa: Tumor Necrosis Factor-alfa
VEGF: Vascular Endothelial Growth Factor
WHO: World Health Organization
